# Supplementary material for: Application of minimum error entropy unscented Kalman filter in table tennis trajectory prediction
Source: PLoS One. 2022 Sep 30;17(9):e0269257. doi: 10.1371/journal.pone.0269257 (PMC9524663; doi:10.1371/journal.pone.0269257)
Supplement: Attachment — Submitted filename: Response to Reviewers.docx [file pone.0269257.s001.docx]

Response to Reviewers

Dear Editor

First, we would like to thank you and the reviewers for your efficient handling of our original paper. The attached manuscript is a revision of our submitted paper entitled “Application of Minimum Error Entropy Unscented Kalman Filter in Table Tennis Trajectory Prediction”. We have modified the manuscript as required to the reviewer comments. Our point-by-point replies to the comments are given below.

**Reviewer #1:**

**comment** **1:** In general, punctuation marks need to be marked at the end of the formula, and the variable needs to be interpreted where it first appears.

Thank you for this comment. This comment can be divided into two questions. Question 1: punctuation marks need to be marked at the end of the formula; question 2: the variable needs to be interpreted where it first appears.

For the first question, the punctuation of these formulas (Eqs. (5), (6), (7), (8), (10), (11), (13), and (18)) is added in the corresponding parts, and the exact modifications are presented in the manuscript.

For the second question, some variables are interpreted where it first appears, such as observation vector $\mathbf{z}_{k}$ and time interval $\Delta t$. Moreover, the other variables have been explained in their first appearance.

**comment** **2:** For a more formal expression, “ping pong ball” should be modified to “table tennis”.

This comment is very meaningful for improving the quality of this manuscript. We have modified all the "ping pong ball" to "table tennis" in the manuscript.

**Reviewer #2:**

**comment** **1:** An algorithm flowchart should be given to better express the subject of the article, and more simulations need to be discussed.

Thank you for this comment. This comment can be divided into two questions. Question 1: an algorithm flowchart should be given to better express the subject of the article; question 2: more simulations need to be discussed.

For the first question, we have added pseudo-code for the algorithm in the modified manuscript, and detailed results are shown below:

For the second question, an additional noise, called Rayleigh noise, is utilized to verify the performance of the algorithm. The corresponding convergence curves and necessary discussion are presented in Fig. 2(b) and manuscript, respectively.

Figure 2: Convergence curves under Rayleigh noise.
